# The implementation and evaluation of an e-Learning training module for objective structured clinical examination raters in Canada

**DOI:** 10.3352/jeehp.2018.15.18

**Published:** 2018-08-06

**Authors:** Karima Khamisa, Samantha Halman, Isabelle Desjardins, Mireille St. Jean, Debra Pugh

**Affiliations:** 1 Division of Hematology, The Ottawa Hospital, Ottawa, ON, Canada; 2 Division of General Internal Medicine, The Ottawa Hospital, Ottawa, ON, Canada; 3 Department of Family Medicine, University of Ottawa, Ottawa, ON, Canada; Hallym University, Korea

**Keywords:** Educational assessment, Undergraduate medical education, Clinical clerkship

## Abstract

Improving the reliability and consistency of objective structured clinical examination (OSCE) raters’ marking poses a continual challenge in medical education. The purpose of this study was to evaluate an e-Learning training module for OSCE raters who participated in the assessment of third-year medical students at the University of Ottawa, Canada. The effects of online training and those of traditional in-person (face-to-face) orientation were compared. Of the 90 physicians recruited as raters for this OSCE, 60 consented to participate (67.7%) in the study in March 2017. Of the 60 participants, 55 rated students during the OSCE, while the remaining 5 were back-up raters. The number of raters in the online training group was 41, while that in the traditional in-person training group was 19. Of those with prior OSCE experience (n= 18) who participated in the online group, 13 (68%) reported that they preferred this format to the in-person orientation. The total average time needed to complete the online module was 15 minutes. Furthermore, 89% of the participants felt the module provided clarity in the rater training process. There was no significant difference in the number of missing ratings based on the type of orientation that raters received. Our study indicates that online OSCE rater training is comparable to traditional face-to-face orientation.

## Introduction

In the context of objective structured clinical examinations (OSCEs), raters are typically provided with an orientation to ensure familiarity with the rating instruments used and to define standards for acceptable performance [1]. There is good evidence that providing structured rater training is helpful in ensuring that raters understand their role, but no consensus exists on who the ideal rater is or the best way to train them [2]. Limited research has been conducted on the use of asynchronous OSCE rater training in the undergraduate curriculum, although a prior study examined the use of an e-Learning resource to enhance rater confidence. In that study, raters were asked to watch up to 12 videotaped simulated OSCE stations and then anonymously compared their scores on checklists and global rating scales to those of others [3]. Global rating scales differ from checklists in that they aim to assess performance as a whole, rather than based on individual components [1]. However, in that study, there was no way to gauge rater performance with respect to data completeness during an actual OSCE.

At the University of Ottawa, we use resident physicians and faculty physicians as raters for undergraduate medical student OSCEs. Raters receive an in-person orientation prior to each OSCE to ensure that they understand their required tasks (e.g., assessing students and/or providing feedback in formative OSCEs). One of the challenges with in-person orientations is that raters have conflicting clinical duties that prevent their attendance; furthermore, they may miss important aspects of such training. To address this issue, we developed an online rater training module for an undergraduate OSCE (Supplement 1).

The purpose of this study was to compare the utility, feasibility, and efficacy of online OSCE rater training and a traditional in-person orientation for raters who assessed third-year medical students during an OSCE at the University of Ottawa, Canada.

## Methods

### Ethical statement

We obtained approval from the Ottawa Health Science Network Research Ethics Board for this study (IRB approval no., OHSNREB #20160756-01H). Written consent was obtained from the study subjects.

### OSCE format

A mandatory, formative, 10-station OSCE was administered to third-year medical students in March 2017 at the University of Ottawa, Canada. The OSCE was composed of a variety of station types (i.e., history-taking, physical examination, communication, and management). Physician raters objective the interactions, assessed candidates’ performance using standardized instruments, and provided verbal feedback.

### Participants

An e-mail invitation was sent to 90 OSCE raters to participate in the study. The physicians recruited were either faculty or senior residents (at least in their third year of post-graduate training). We allocated study participants to receive their orientation either through the online module or through the traditional in-person session. Allocation was by random number assignment (2:1 for online training vs. in-person). However, raters who volunteered within 24 hours of the OSCE were automatically allocated to the in-person group to ensure that they would have time to be oriented.

### Module development

We created an online training module to provide an orientation for physician OSCE raters (Supplement 1). The module was developed in French and English, given the bilingual nature of the University of Ottawa.

### Administration

For the online group, we asked raters to complete the module up to 1 week before the OSCE. They were able to progress through the module at their own pace, and to complete it in more than 1 sitting if desired. We administered a 10-question multiple-choice quiz following completion to verify that they understood the content. We provided immediate written corrective feedback for any incorrect answers. We tracked participation, and were able to confirm that participants had completed the module. For those allocated to the in-person group, study investigators presented a 30-minute didactic orientation (Supplement 2). A research assistant noted the arrival time for any late raters.

### Rating scale completeness

For each OSCE station, we asked raters to complete 3 instruments: (1) a case-specific checklist; (2) between 3 and 7 rating scales (e.g., rapport, organizational skills); and (3) a 6-point global rating. For checklist items, raters either provided a checkmark (for items that were done satisfactorily) or left it blank (for items that were not done satisfactorily or not attempted at all). For the rating scales and global rating, it was mandatory that raters provided a score for each item. Sample rating scales and global rating scales are attached (Supplement 2, page 11–13). Checklists were kept confidential, as cases are used in future years.

Following the administration of the OSCE, we calculated the percentage of completeness for all rating scales and global ratings. As there was no option for a blank score on the rating scales or global rating, any blank items were treated as missing data. It was not possible to calculate data completeness on checklists, as blank items could represent items not attempted by the candidate, items unsatisfactorily performed by the candidate, or items missed by the rater. We used the t-test to analyse differences in rating scale completeness between the 2 groups (online and in-person orientation).

### User satisfaction

We sent a short online survey (Fluid Surveys) to all study participants following the OSCE to get feedback on their experience (Supplement 3). We used the Mann-Whitney U-test to evaluate differences between the groups in the proportion of raters who were faculty physicians versus residents, and the proportion of raters invigilating their first OSCE versus those with prior OSCE experience. We used the 2-sided t-test to explore differences in rater confidence in performing their role depending on which type of orientation they received. We used partial eta squared to determine the effect size. We reviewed narrative comments to identify areas for improvement in our rater orientation. To incentivize survey completion, we held a drawing for an iPad mini 4.

## Results

A total of 90 physicians were invited to participate, of whom 60 consented to be part of the study (67.7%). Forty-one raters were allocated to the in-person orientation (including those non-randomly allocated in the final 24 hours before the OSCE), and 19 were allocated to the online orientation. Data from the English-speaking (38) and French-speaking (3) raters were combined. Five of those who consented did not actually participate in the OSCE, as they were back-up raters. Thus, data from 55 raters (15 from the online group and 40 from the in-person group) were available for the analyses of rating scale completeness.

### Demographics

There was no significant difference in the proportion of faculty and residents allocated to the online and in-person groups (P= 0.897), nor was there a significant difference in experience (P= 0.987) (Supplement 4).

### Satisfaction

Table 1 presents the results of the post-administration survey with regards to length of presentation, clarity of content, and interactivity of the presentation. The majority of raters in the online orientation group evaluated the length, clarity, and interactivity of the presentation as excellent (proportionally higher than in-person group).

Those in the online group (mean= 3.74, standard deviation [SD]=0.452) provided significantly higher evaluations (F[1]=10.56, P=0.002, partial eta squared= 0.154) when asked about their confidence with the rating task than those in the in-person group (mean= 3.32, SD=0.471). Of the raters who completed the in-person orientation, 100% claimed that they were present for the entire orientation, when in fact 8 were late (mean= 10 minutes). The average time needed to complete the e-Learning module was 15 minutes (n= 16). Not all participants were able to estimate the time needed to complete the module. Study participants noted no major login/technical issues with the module; the written comments reflected a high level of satisfaction with the online training (Supplement 4).

### Rating scale completeness

There were 62 mandatory ratings that were left blank. There was no significant difference in the number of missing ratings based on the type of orientation that raters received (online group: mean= 0.87 versus in-person group: mean= 1.23; P= 0.444). Additionally, when the non-randomized latecomers were removed from the analysis, there was no significant difference in the number of missing ratings based on the type of orientation that examiners received (online group: mean= 0.87 versus in-person group: mean= 0.75; P= 0.794).

Prior objective structured clinical examination rater experience

Of those with prior OSCE experience (n= 18) who participated in the online orientation (n= 41), 13 (68%) reported that they preferred this format to the in-person orientation.

## Discussion

The convenience and flexibility of an online format for OSCE raters was appealing across a spectrum of experiences. High satisfaction rates were noted with respect to clarity, length of presentation, and interactivity of the online module compared with traditional face-to-face training (Table 1). Eight raters arrived late for the faceto-face orientation, potentially compromising their ability to perform the required rating tasks. This is not an uncommon occurrence for busy clinicians who serve as raters.

A unique aspect of the online training was the requirement for raters to obtain 100% on the end-of-module quiz (Supplement 1). Studies have shown that this type of assessment can enhance learning and retention [4]; in contrast, the in-person training had a traditional didactic presentation with more passive learning.

The study was not without limitations. The online module was limited to orienting raters to the tasks required for a formative OSCE, but did not include frame-of-reference training (e.g., videos that present performance differences between candidates). As well, while those undergoing online training showed improved confidence in their rating tasks when compared to those undergoing face-to-face training, we cannot determine whether this increase in confidence led to improved accuracy, as data completeness was equivalent in both groups.

As well, there may be unintended consequences to this strategy in the future, as raters may arrive even later than anticipated. Additionally, information technology support must be in place to support this strategy; this may limit implementation at some institutions.

In conclusion, our study suggests that online OSCE rater training is feasible and comparable to in-person training for clinicians. This was a preliminary study at a single centre. Further studies using multiple different sites are needed to support a wider role for online training of OSCE raters.

## Figures and Tables

**Table 1.** Participant satisfaction with orientation

Survey item	In-person orientation ratings (n = 41)			Online orientation ratings (n = 19)		
	Poor	Adequate	Excellent	Poor	Adequate	Excellent
Length of presentation	1 (2)	23 (56)	17 (41)	1 (5)	2 (10)	16 (84)
Clarity of content	0	20 (49)	21 (51)	0	1 (5)	17 (89)
Interactivity of presentation	0	26 (63)	15 (36)	0	1 (5)	17 (89)

Values are presented as number (%).

## Supplementary materials

Supplement 1. Storyboard online OSCE rater training module.

Supplement 2. In-person OSCE Rater training presentation.

Supplement 3. Participant survey tool.

Supplement 4. Data files are available from https://doi.org/10.7910/DVN/RBL9MV

Supplement 5. Abstract recording.
