# Supplementary material for: The implementation and evaluation of an e-Learning training module for objective structured clinical examination raters in Canada
Source: J Educ Eval Health Prof. 2018 Aug 6;15:18. doi: 10.3352/jeehp.2018.15.18 (PMC6194479; doi:10.3352/jeehp.2018.15.18)
Supplement: Supplementary file 1 — Supplement 1. Storyboard online OSCE rater training module. [file jeehp-15-18-suppl1.pdf]

Storyboard

PRJ0011946 – OSCE Examiner Online Orientation

Client: UGME
Subject Matter Expert (SME): Dr. Karima Khamisa
uOttawa Faculty of Medicine
Instructional Designer (ID): Christina Desormeaux, Medtech

Revision History

Version	Date (yyyy-mm-dd)	Author	Description
1	2017-12-08	Christina Desormeaux	Initial Draft
2	Insert Date	Insert Name	SME Review draft 1
3	Insert Date	Insert Name	Changes integrated
4	Insert Date	Insert Name	SME final review
5	Insert Date	Insert Name	Changes integrated
6	Insert Date	Insert Name	SME approval /sign off

Table to Contents

About this document 4

Home 5
- Learning Objectives 5
- Timing 5
- Module Navigation 5
- Successful completion 5
- Getting Help 6

OSCE Overview 6
- What are the OSCEs? 6
- Responsibilities 7
- Conflicts of Interest 8
- Types of Stations 9

Before the Examination 10
- Sign in 10
- Conduct a room check 10
- Conduct a dry run 10
- Review the scoring guidelines and checklist for your case 11

During the Examination 11
- Be aware 11
- Take note of timing 11
- Provide Prompts and ask questions 12
- Score the candidate using rubrics 13
- Provide feedback 15

After the Examination 15

Quiz 17
- Instructions 17
- Questions 17

Next Steps **20**
Credits **20**
Resources **21**
References **21**

About this document

The storyboard captures all the content that will be covered in the learning module including images, tables and other content.

In general, one screen represents all the content for that topic/subtopic.

Use the **Track Changes** and **Comments** feature to discuss and communicate changes. If more than one person is reviewing this document, all comments must be consolidated into one storyboard.

Do not modify the content in the Notes to the Developer column.

Onscreen instructions to learners will be in green

Directions the developer that appear in the Onscreen Text column will be [in brackets and orange]

Links to sites or a pop-up with additional information will be in blue and underlined

#	Main Screen Topic/Title	On-Screen Content	Notes to Developers
1	**Home**	The validity of scores obtained from objective structured clinical examinations (OSCEs) is dependent on the quality of data that is collected from examiners. To ensure that OSCE scores accurately represent students' abilities, it is imperative that examiners are completing rating instruments correctly.<br><br>The purpose of this eLearning module is to provide you, the OSCE examiner with the knowledge and skills necessary to correctly conduct OSCE examinations.<br><br>**Learning Objectives**<br>After completing the modules, you should be able to:<br>• Provide an overview of the OSCE including its purpose and its target audience<br>• Describe OSCE examiner responsibilities<br>• Explain what to expect before an examination<br>• Describe effective interactions with candidates (i.e., prompts and oral questions)<br>• Use scoring rubrics (i.e., checklists, rating scales, global judgement)<br>• Describe effective feedback (i.e., written and verbal)<br>• Explain the importance of data completeness<br>**Timing**<br>This module will take about 30 minutes to complete<br><br>**Module Navigation**<br>You will be able to navigate to any module/topic and complete them at your own pace. If you exit the module before completion, your page will be bookmarked so that you can easily resume learning from where you left off during the previous session. You can repeat the module as often as needed.<br>**Successful completion**<br>Successful completion of this module requires you to:<br><br>• Complete a quiz at the end with a score of 100%	Home page for ipad (style tbd)

#	Main Screen Topic/Title	On-Screen Content	Notes to Developers
		• Complete an evaluation survey of this modules<br><br>This module must be completed by March 4th, 2017<br><br>**Getting Help**<br>• Module Content Support – xxx<br>• Technical Support – Medtech	
2	**OSCE Overview**	**What are the OSCEs?**<br>[insert image of OSCE in progress]<br><br>The OSCE is a formative examination that is mandatory for students in the third year of medical school training at the University of Ottawa. At this point, students have successfully completed the first two years of pre-clerkship training and have completed at least **6 months** of clinical clerkship training. NOTE: **Not** all rotations have been completed.<br><br>The purpose of the OSCE is to test their progress. Students will be taking their OSCE on March 6th, 2017. This is in preparation for the "high stakes" Comprehensive OSCE - at the beginning of their 4th year.<br><br>You are required to evaluate candidate performance and mark exam sheets accordingly.<br><br>You are responsible to ensure that the scoring of performance is **fair and valid** for all candidates.<br><br>**Students are expecting to receive feedback at the end of each station by an examiner**.<br><br>[Popup begin]<br><br>**Formative Examination**<br><br>A formative examination is meant to assess a student's progress and is on	**Image:** OSCE in progress

#	Main Screen Topic/Title	On-Screen Content	Notes to Developers
		opportunity for a supervisor to provide immediate feedback to improve performance<br><br>[Popup end]	
3	**OSCE Overview**	**Responsibilities**<br><br>The OSCE Examiner has responsibilities regarding arrival time, appropriate attire and the use of electronic devices.<br><br>Click each marker to learn more.<br><br>[Insert image of examiner]<br><br>Marker 1 pointing to watch<br>**Arrival Time**<br>We acknowledge that physician examiners have clinical duties prior to evening OSCEs.<br><br>We would request that examiners arrive on time; any unexpected delays or absences should be communicated to The Ottawa Exam Centre by sending an email examctr@uottawa.ca.<br><br>Marker 2 pointing to clothing<br>**Appropriate Attire**<br>We would request a minimum dress code of business/business casual. Scrubs are acceptable. We would ask examiners to refrain from wearing (add info graphic):<br>• Ripped jeans<br>• Hoodies<br>• Jogging pants<br>• T shirts or shorts<br><br>Please avoid wearing scented products on the day of the exam.	**Interaction: Labelled image with pop-ups**<br>Show **image of an examiner** wearing a watch, proper clothing and holding a phone. Provide clickable markers pointing to each of these elements.<br><br>Learner clicks marker and popup with details appear.

#	Main Screen Topic/Title	On-Screen Content	Notes to Developers
		Marker 3 pointing to cell phone<br>**Use of Electronic Devices**<br>Please refrain from using any electronic devices during the examination; all pagers, iPads, laptops and other devices must be set to SILENT mode.<br><br>Only answer calls during breaks. Physicians who are on call, should not volunteer to be examiners.	
4	**OSCE Overview**	**Conflicts of Interest**<br>If you perceive a potential conflict of interest with a candidate, please terminate the encounter and notify staff immediately.<br><br>The student will then be permitted to complete the station with a different examiner at the end of the exam.<br><br>***Which of the following would you consider a conflict of interest?***<br>Select all that apply.<br><br>☐ The candidate is my sister<br>☐ The candidate is a former patient of mine<br>☐ The candidate is a former girlfriend of mine<br>☐ The candidate is my roommate's girlfriend<br>☐ I have a financial/business relationship with the candidate<br>☐ I worked with the candidate during a core rotation<br><br>[Insert *Compare your answer* button]<br><br>**Correct Answer**<br>☑ Candidate is a family member<br>☑ Candidate is (or has been) a patient of yours<br>☑ You are (or have been) in a romantic relationship with the candidate<br>☑ You know the candidate<br>☒ You have a financial/business relationship with the candidate	**Interaction:** Question with multiple choice multiple answer response and pop-up feedback.<br><br>The answer does not have to be tracked. When the user clicks *Compare your answer*, highlight the correct answers and their checkmarks so they are green.

Commented [CD1]: Please provide an example of a plausible financial/business relationship

#	Main Screen Topic/Title	On-Screen Content	Notes to Developers
		☒ You have worked with the candidate (e.g., during a core rotation or elective)<br>[Lightbox end]	
5	OSCE Overview	Types of Stations<br>When you arrive, you will be assigned to one of four stations.<br><br>Select each station to learn more.<br><br>Tab 1 Title and Content<br>**History taking**<br>A candidate is required to take a focused history relevant to the problem.<br><br>Tab 2 Title and Content<br>**Physical examination stations**<br>A candidate must demonstrate and vocalize the pertinent clinical examination techniques relevant to the problem. Candidates must "Talk the talk" and "walk the walk".<br><br>**Note: No sensitive examinations are permitted–** if attempted, indicate "NOTED, MOVE ON"<br><br>Tab 3 Title and Content<br>**Management stations**<br>The candidate must take a focused history and perform a brief physical examination relevant to the problem AND provide instructions for relevant investigations and preliminary orders in an emergent setting.<br><br>Tab 4 Title and Content<br>**Counselling and Communication stations**<br>Candidates must build a therapeutic alliance with the patient and demonstrate knowledge of pharmacologic and non-pharmacologic interventions required to manage a patient with a particular condition	**Interaction: Tabs with content**<br>Learner clicks each tab and image with information about the station appears above.

#	Main Screen Topic/Title	On-Screen Content	Notes to Developers
	Before the Examination	**Sign in**<br>✓ Ensure you have signed in at the registration desk<br>✓ Complete paperwork for payment signed and collect sticker indicating Track and Station.<br>✓ Dinner will be provided at 5:15 pm. During dinner, feel free to ask questions to clarify any aspect of the exam<br>✓ Ottawa Exam Centre Staff will escort you to the correct track and station<br><br>[Insert image of the exam centre staff person]	**Image:** Exam centre staff person
6		**Conduct a room check**<br>Once you are in your correct room.<br><br>✓ Review the equipment and props for the case<br>✓ Report any missing props or faulty equipment to the Exam Centre staff (e.g. malfunctioning blood pressure cuff)<br><br>[insert image of the props]	**Image:** Examples of equipment and props
7	Before the Examination	**Conduct a dry run**<br>Introduce yourself to the standardized patient (SP) and conduct a dry run.<br><br>***Why do you think it's important to conduct a dry run?***<br><br>[insert open textbox]<br><br>[Insert *Compare your answer* button]<br><br>**Feedback**<br><br>Conduct a dry run with the SP:<br><br>• This dry run will help both you and the SP to prepare for the first candidate<br>• It will also help you define what is a satisfactory candidate	**Interaction:** Question with open-text response.<br><br>Learner can type in answer then click *Compare your answer* button to view the correct answer.

#	Main Screen Topic/Title	On-Screen Content	Notes to Developers
		response/behaviour for each checklist item	
8	**Before the Examination**	**Review the scoring guidelines and checklist for your case**<br>✓ Oral questions?<br>✓ Findings to report?<br>✓ Alert the Ottawa Exam Centre staff if you have a question relating to the checklist. We can often answer before exam starts.<br>✓ Do not change the case or checklist during the exam– needs to be standardized throughout exam<br>✓ Use the comment sheet in each station to alert us to the error:<br>o We can adjust scoring for all candidates, in a standardized way<br>o We will revise the case for the next time it is used<br>o Write us a new (and better) case for next year!<br><br>[Insert image of scoring guidelines and checklist]	**Image:** Scoring guidelines and checklist (provide a pdf) with example of case.
9	**During the Examination**	**Be aware**<br>Keep voices quiet between candidates<br>[insert image of the person]<br><br>Standardized patients may change during the exam – don't be surprised if a new patient enters the room halfway through.<br>[insert image of the person leaving room]	**Image:** People talking quietly or silence sign<br><br><br>**Image:** Person leaving room while another is entering
10	**During the Examination**	**Take note of timing**<br><br>Each station is 9 minutes long. You must be aware of what to do at each interval.<br><br>Select each time interval to learn more.<br><br>Marker 1 pointing to 0<br>**0 minute**<br>You will hear the long buzzer. This is the start. Enter the room and begin the	**Interaction: Labelled image with pop-ups**<br><br>Show image of a timer with markers at each time of importance. Learner clicks marker and details of each timing appears.

#	Main Screen Topic/Title	On-Screen Content	Notes to Developers
		encounter<br><br>Marker 2 pointing to 7 minutes<br>**7 MINUTES**<br>You will hear the Intermittent buzzer. Stop, ask the oral question (if there is one) and provide feedback.<br><br>Marker 3 pointing to 9 minutes<br>**9 MINUTES**<br>You will hear the long buzzer. Leave the room and move to the next station. Read the instructions.<br><br>Marker 4 pointing to 10 minutes<br>**10 MINUTES**<br>You will hear the long buzzer. Enter the room and begin the next encounter.	Long buzzer **link:** Opens video of a close up hand pushing pressing long buzzer. Include audio of buzzer.<br><br>Intermittent buzzer **link:**<br>Opens video of a close up hand pushing pressing intermittent buzzer. Include audio of buzzer.
11	**During the Examination**	**Provide Prompts and ask questions**<br><br>Tab 1 Title and Content<br>**Provide prompts**<br><br>***Can you think of any circumstances in which it would be acceptable to intervene with a standardized patient?***<br><br>[Insert *Compare your answer* button]<br><br>You many intervene when the standardized patient needs help e.g. manoeuvre causes unnecessary pain, is invasive or inappropriate. Candidates should never perform rectal or genital examinations. If they talk about their intention to perform such examinations, tell the candidate “noted, please move on” and score as satisfactory.<br><br>***Can you think of any circumstances in which it would be acceptable to intervene***	**Interaction: Tab and content**<br><br>Learner clicks tab title and information/activity within appears.<br><br>When the learner clicks the *Compare your answer* button, the answer should appear below the question.<br><br>When the learner clicks the *Compare your answer* button, the answer

#	Main Screen Topic/Title	On-Screen Content	Notes to Developers
		with a candidate?<br><br>[Insert *Compare your answer* button]<br><br>**You may also intervene when a candidate has misunderstood the task**, e.g. starts taking a history in a physical exam station or vice versa. Instruct him/her as follows:<br>• Please re-read the instructions<br>• You are allowed to re-direct the candidate ONCE. For example, when at the history station, the student starts doing a physical exam. The examiner should state, "please read the instructions again". This is the only acceptable prompt<br>• Please do not offer any other prompts even if the student is struggling. For example, do not redirect them if they start doing vital signs, unless it is specifically mentioned on your checklist<br><br>Tab 2 Title and Content<br>**Ask oral question when required**<br>Some stations will require you to ask one or more oral question(s) to the candidate:<br>• Ask AFTER the intermittent buzzer<br>• Candidates CANNOT continue interacting with the standardized patient after the intermittent buzzer<br>• Read the question (s) as written, DO NOT improvise<br>• Only repeat if the candidate specifically requests it<br>• Please indicate the answer the candidate provides<br>• Not every station has a post-encounter question; in that case you can use the entire 2 minutes for feedback	should appear below the question.
12	During the Examination	Score the candidate using rubrics<br>When the candidate enters the room please ensure they provide you a [marker 1] barcode sticker that should be placed in the top right corner of the scoring sheet.<br><br>You are responsible for completing **BOTH sides of the scoring sheets** that contain:	**Interaction: Labelled image with pop-ups**<br>Show **image of Examiner Checklist** (2 pager). Place markers over each section. Learner clicks the marker and pop-up appears with more details.

#	Main Screen Topic/Title	On-Screen Content	Notes to Developers
		• Station checklist<br>• A professionalism question<br>• Rating scales<br><br>Select each type of scale to learn more about the correct way to fill in the scanned items.<br><br>Marker 1 pointing to section on Rating Scales<br>**Rating Scales**<br>Station specific skills set (e.g. history taking, physical examination, communication)<br><br>A global rating of the candidate performance relative to a THIRD YEAR medical student<br><br>Marker 2 pointing to Professionalism question<br>You will also be asked to answer the following question:<br><br>**Did this candidate demonstrate a lapse in professional behaviour?**<br><br>• If you answered YES, you must describe<br>• Examples include – being rude or dismissive to patient; inappropriate draping during physical examination or candidate wearing inappropriate attire<br><br>Marker 3 point to section on Global Rating Scale<br>Use your judgment to rate each candidate’s overall performance (remember, they are 3rd year students).<br><br>A satisfactory performance does not depend on the number of checklist items scored correctly.<br><br>Your judgments define the borderline candidate and are used to set the pass mark for each station.	

#	Main Screen Topic/Title	On-Screen Content	Notes to Developers
		If you rate a candidate as unsatisfactory, briefly indicate your reasons on the comment sheet (this helps when we review with students).<br><br>An unsatisfactory rating is not equivalent to a fail. Examples include:<br><br>• Ordered dangerous or inappropriate drug (specify drug)<br>• Insensitive manner (examples and quotes especially helpful)<br>• Would not / could not interact with patient<br>• Rote performance - did not demonstrate comprehension of patient problem ("shotgun approach")	
13	**During the Examination**	**Provide feedback**<br>This is a FORMATIVE OSCE. Feedback MUST be provided to the student on any aspect of their performance. Give feedback only after intermittent buzzer and post-encounter question:<br>• Provide feedback on anything you feel appropriate such as physical exam technique, important items they missed, patient interaction, organization skills, etc.<br>• Balance positive with negative<br>• Ask the standardized patient their opinion (especially in communication stations)<br><br>DO NOT INDICATE TO THE CANDIDATE WHETHER THEY "PASSED" OR "FAILED" A STATION; this will be determined after standard setting has been completed.	
14	**After the Examination**	After the examination, remain in room until the scoring sheets are collected.<br><br>Select each tab for additional post-examination details.<br><br>**Panel 1**<br><br>**Feedback sheets**<br><br>Please complete the feedback sheets provided regarding content of the cases,	**Interaction:** Accordion with content in panels.

<table>
<tr><th>#</th><th>Main Screen Topic/Title</th><th>On-Screen Content</th><th>Notes to Developers</th></tr>
<tr><td></td><td></td><td>
standardized patient portrayal and any other feedback from the evening.

Panel 2

Examination Schedule

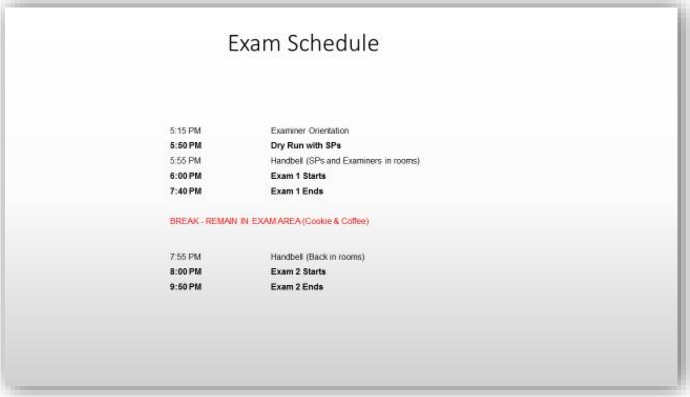

Panel 3

Remuneration

Thank you for your involvement as an Examiner for the OSCE

Faculty/staff will be paid $500 for the evening, and should expect payment within 6-8 weeks from the University

** Faculty from the DOM will be paid through STAR
</td><td></td></tr>
</table>

#	Main Screen Topic/Title	On-Screen Content	Notes to Developers
		• Please complete the Physician Examiner Invoice and HST Declaration form<br><br>Panel 4<br><br>**Spare Examiners**<br><br>Thank you for coming!<br><br>Exams cannot function without availability of spare examiners<br><br>Paid $250 for evening<br><br>Please stay in auditorium until exam has started (usually about 30 minutes) – chief examiner will return to let you know whether you are needed or not.	
14	**Quiz**	**Instructions**<br>Thank you for completing the online module. You will now be required to complete a quiz. All questions must be answered correctly to complete the module.<br><br>**Questions**<br><br>**Question 1**<br>***Which of the following is appropriate attire ?***<br>Select all that apply.<br><br>☐ **Scrubs**<br>☐ Ripped jeans<br>☐ **Suit jacket and pants**<br>☐ Hoodie<br>☐ Jogging pants<br>☐ T shirts and shorts<br><br>**Correct answer feedback:** That's right!<br>**Incorrect answer feedback:** Please try again	

Commented [CD2]: Questions should at minimum test each learning objectives. I notice that 3/8 cover one (OSCE examiner responsibilities.

Commented [KK3]: Good point! Okay we can look at this

Commented [CD4]: Can you provide additional examples of appropriate wear?

Commented [CD5]: Insert more feedback to reinforce the response and review content.

Commented [KK6]: Good idea – I like "That's Right"

#	Main Screen Topic/Title	On-Screen Content	Notes to Developers
		**Question 2**<br>What do you do if the candidate is a family member or you have a close personal relationship with this person?<br>○ Allow the candidate to proceed with the exam<br>○ Leave the room<br>○ **Stop the encounter and alert one of the staff that there is a potential conflict**<br>○ All of the above<br><br>**Correct answer feedback:** That's right!<br>**Incorrect answer feedback:** Please try again<br><br>**Question 3**<br>What do you do if you know a student because they have recently completed a rotation with you?<br>☐ **Allow the candidate to proceed with the exam**<br>☐ Leave the room<br>☐ Stop the encounter and alert one of the staff that there is a potential conflict<br>☐ All of the above<br><br>**Correct answer feedback:** That's right!<br>**Incorrect answer feedback:** Please try again<br><br>**Question 4**<br>What do you do if you detect a problem with the checklist?<br>☐ Make a note on the comment sheet provided and change the checklist to ensure it is the way you like it<br>☐ **Make a note on the comment sheet, but DO NOT change the checklist.**<br>☐ Advise the student the checklist is wrong<br>☐ Advise the student to stop the examination	

#	Main Screen Topic/Title	On-Screen Content	Notes to Developers
		**Correct answer feedback:** That's right!<br>**Incorrect answer feedback:** Please try again<br><br>**Question 5**<br>Do you give feedback to the candidate on their performance during the Teaching and Testing OSCE?<br>☐ **Yes**<br>☐ No<br><br>**Correct answer feedback:** That's right!<br>**Incorrect answer feedback:** Please try again<br><br>**Question 6**<br>What is the ONLY acceptable PROMPT that can be given to a candidate if they start taking a history during a physical examination station?<br>☐ Please stop the examination<br>☐ Please re-do the whole station<br>☐ Please read the instructions again<br>☐ No prompting is allowed; remain silent throughout the station<br><br>**Correct answer feedback:** That's right! They can redirect ONCE only.<br>**Incorrect answer feedback:** Please try again<br><br>**Question 7**<br>Are students allowed to perform sensitive examinations (rectal, genital or breast examinations) during this OSCE?<br>☐ Yes<br>☐ **No**<br><br>**Correct answer feedback:** That's right! Students are not permitted to perform sensitive examinations. There are very firm instructions will confirm this and will elaborate that the examiner must say NOTED, MOVE ON<br>**Incorrect answer feedback:** Please try again	

Commented [CD7]: Insert more feedback to reinforce the response and review content.

Commented [CD8]: What is the Teaching and Testing OSCE? What learning objective does this cover?

Commented [CD9]: Insert more feedback to reinforce the response and review content.

Commented [CD10]: Add more here? This is a great opportunity to review important teaching points.

Commented [CD11]: This sentence is awkward. Why not take this opportunity to repeat the instructions in full?

#	Main Screen Topic/Title	On-Screen Content	Notes to Developers
		**Question 8**<br>What do you do if a student starts to do vital signs?<br>☐ **Let them proceed, unless the checklist instructs you do to otherwise**<br>☐ Tell them to stop, as this will waste time<br>☐ Tell them that the patient is stable and move on<br>☐ Tell them the vital signs at the beginning of each station<br>**Correct answer feedback:** That's right!<br>**Incorrect answer feedback:** Please try again	
15	**Next Steps**	Well done! You have successfully completed the **OSCE Examiner Online Orientation.**<br><br>For additional information, please take the time to visit the resources and references tabs of this module.<br><br>To receive full credit for this module, please complete the Module Evaluation Survey. Your feedback will help improve this learning experience for others.<br><br>Thank you.	
16	**Credits**	**Created by:** xxx, University of Ottawa<br>**Subject Matter Experts:**<br>Insert Name (First Last)<br>Insert Credentials (e.g. PhD, MD, FRCPC)<br>Insert Division<br>Insert Department<br><br>**Editorial Committee (or Peer Review?)**<br>Insert Name (First Last)<br>Insert Credentials (e.g. PhD, MD, FRCPC)<br>Insert Division<br>Insert Department	

Commented [CD12]: Insert more feedback to reinforce the response and review content.

#	Main Screen Topic/Title	On-Screen Content	Notes to Developers
		**Instructional Design and Production** - Medtech eLearning, University of Ottawa	
17	**Resources**	List resources that the learner can access to support learning and to learn more	
18	**References**	List resources that the learner can access to support learning and to learn more	
