# Supplementary material for: The implementation and evaluation of an e-Learning training module for objective structured clinical examination raters in Canada
Source: J Educ Eval Health Prof. 2018 Aug 6;15:18. doi: 10.3352/jeehp.2018.15.18 (PMC6194479; doi:10.3352/jeehp.2018.15.18)
Supplement: Supplementary file 2 — Supplement 2. In-person OSCE Rater training presentation. [file jeehp-15-18-suppl2.pdf]

Teaching and Testing OSCE

Examiner Orientation

Chief Examiner - Anglophone: Dr. Karima Khamisa

Chief Examiner - Francophone: Dr. Samantha Halman

Teaching and Testing OSCE

- Third year medical students
- 1/2 finished their core clerkship rotation year
  - NOTE: not all their rotations completed
- Formative Exam
  - Tests their progress
  - Receive feedback at the end of each station by examiner
  - Preparation for the “high stakes” Comprehensive OSCE

Types of Stations

- History taking
- Physical examination
  - Students must explain what they are doing, demonstrate the skills and state their findings
  - “Talk the talk” and “walk the walk”
  - No sensitive examinations – if attempted, indicate “MOVE ON”
- Management
  - Checklist includes items for history-taking, physical exam and management
- Counselling and Communication

Prompting

- You are allowed to redirect the student **once**
  - e.g., history station and student starts doing a physical exam
  - Examiner should state **“please read the instructions again”**
  - *This is the only acceptable prompt*
- Please **do not offer any other prompts** even if the student is struggling
  - e.g., do not redirect them if they start doing vital signs, unless it is specifically mentioned on your checklist

Feedback

- Give **feedback** only **after buzzer and post-encounter question**
- Provide give feedback on anything you feel appropriate
  - Physical exam technique, important items they missed, patient interaction, organization skills, etc.
  - Balance positive with negative
  - Ask the standardized patient their opinion (especially in communication stations)

Checklists and Rating Scales

What to do if you think there is an error in the checklist or case ?

- Alert exam staff (in red t-shirts) if you have a question relating to checklist or case
  - We can often answer **before** exam starts
- Do not change the case or checklist during the exam– needs to be standardized throughout exam
- Use the comment sheet in each station to alert us to the error
  - We can adjust scoring for all candidates, in a standardized way
  - We will revise the case for the next time it is used
- Write us a new (and better) case for next year!

Checklist

- Fill in bubble for item done <u>correctly</u>
  - Leave blank if not attempted
  - Leave blank if attempted but done incorrectly

- If you are not sure – commit and write a comment on sheet explaining your choice
  - Helpful when reviewing examinations for candidates that are unsuccessful

All Scales and Checklists are on Scanned Sheets

Item / Skill	**Done/Asked Satisfactorily**
Introduces self	O
Onset of chest pain	O
Location of pain	O
Radiation	O
Fever	O
Weight loss	O

How to fill in scanned checklists

- This is correct way:

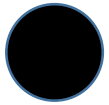

- Wrong way

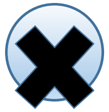

Rating Scales

- **DO NOT FORGET TO TURN SCORING SHEETS OVER**
- In addition to the checklists, there are *three* rating scales for examiners to complete
  - Station specific skills set (history-taking, physical examination, communication)
  - Professionalism
  - Global rating of candidate performance relative to a third year medical student

History-Taking Skills

Listening Skills

0	1	2	3	4	5
Interrupts inappropriately, ignores patient's answers	Impatient	Borderline unsatisfactory Somewhat attentive	Borderline satisfactory Somewhat attentive	Attentive to patient's answers	Consistently attentive to answers and concerns

Questioning Skills

0	1	2	3	4	5
Awkward, exclusive use of leading or closed ended questions, jargon	Somewhat awkward, inappropriate terms, minimal use of open-ended questions	Borderline unsatisfactory, moderately at ease, appropriate language, uses different types of questions	Borderline satisfactory moderately at ease, appropriate language, uses different types of questions	At ease, clear questions, appropriate use of open and closed ended questions	Confident and skillful questioning

Organization of interview

0	1	2	3	4	5
Scattered, shot-gun approach	Minimally organized	Borderline unsatisfactory Flow is somewhat logical	Borderline satisfactory Logical flow	Logical flow with sense of purpose	Purposeful, integrated handling of encounter

Information giving

0	1	2	3	4	5
No attempt or inappropriate attempt to give information; e.g., not truthful	Awkward and/or incomplete attempts to give information	Borderline unsatisfactory Somewhat at ease, attempts to give information	Borderline satisfactory Somewhat at ease, attempts to give information	Gives information easily, somewhat attentive to patient's understanding	Confident and skillful at giving information, attentive to patient's understanding, truthful

Professionalism and Ethics

Professionalism

Empathy

0	1	2	3	4	5
Condescending, not empathetic	Minimal courtesies only	Borderline unsatisfactory	Borderline satisfactory	Polite and interested; some compassion	Warm, engaged with patient; empathetic, and compassionate

Respect

0	1	2	3	4	5
Offensive or aggressive; frank exhibition of “unprofessional conduct”	Minimal courtesies only; not attentive to patient comfort or concerns	Borderline unsatisfactory	Borderline satisfactory	Attentive to patient’s concerns and comfort; some acknowledgment of patient autonomy	Fully addresses patient’s concerns and comfort; fully acknowledges patient autonomy

During the exam

- Completed exam sheets will be collected periodically by exam staff
- Staff will inform you if there are any errors or omissions in your scoring

Professionalism Question

- You will be asked if the candidate demonstrated a lapse in professional behaviour:
  - If you answered yes, please provide a brief explanation in space provided

Global Rating Scale

- Use your judgment to rate each candidate's overall performance (*remember, they are 3rd year students*)
  - not dependent on checklist items
- **Your judgments define the borderline candidate and are used to set the pass mark for each station**
- If you rate a candidate as unsatisfactory, briefly indicate your reasons on the comment sheet (this helps when we review with students)
- An unsatisfactory rating is not equivalent to a fail

Global Rating Scale – Unsatisfactory Performance

- Ordered dangerous or inappropriate drug (specify drug)
- Insensitive manner (examples and quotes especially helpful)
- Would not / could not interact with patient
- Rote performance - did not demonstrate comprehension of patient problem (“shotgun approach”)

Global rating of candidate performance relative to a third year medical student

Unsatisfactory			Satisfactory		
Inferior	*Poor*	*Borderline*	*Borderline*	*Good*	*Excellent*

Comments:

Conflict of Interest

If you perceive a potential conflict of interest with a candidate, please **terminate the encounter** and notify staff immediately

- The student will then be permitted to complete the station with a different examiner at the end of the exam

Conflict of Interest

Examples of conflict of interest

- Candidate is a family member
- Candidate is (or has been) a patient of yours
- You are (or have been) in a romantic relationship with the candidate
- You have a financial/business relationship with the candidate

Conflict of Interest

Not a conflict of interest

- You know the candidate
- You have worked with the candidate (e.g., during a core rotation or elective)

Housekeeping

Exam location

- Clinic “modules”
  - K, L, M, O tracks
- Medical school CBL/PSD rooms
  - H and U tracks
- If you don’t know where you’re going:
  - Exam center staff (in red t-shirt) will escort examiners to medical school – please meet at desk outside auditorium

During exam

- Please do a dry run with the standardized patient prior to the exam
- Please have your pager, IPad and phone turned off or on quiet
- Only answer calls during break
- Keep voices quiet between candidates
- Standardized patients may change during exam – don’t be surprised if new patient enters room half way through

Examiner Remuneration

Spare Examiners

Exam Schedule

5:15 PM	Examiner Orientation
**5:50 PM**	**Dry Run with SPs**
5:55 PM	Handbell (SPs and Examiners in rooms)
**6:00 PM**	**Exam 1 Starts**
**7:40 PM**	**Exam 1 Ends**

BREAK - REMAIN IN EXAM AREA (Cookie & Coffee)

7:55 PM	Handbell (Back in rooms)
**8:00 PM**	**Exam 2 Starts**
**9:50 PM**	**Exam 2 Ends**

Timing

**START= 0 Minute**	Enter the room, begin the encounter
**Long Buzzer**	
**7 MINUTES**	Stop, ask oral question (if there is one)
**Intermittent buzzer**	and provide feedback
**9 MINUTES**	Leave the room
**Long Buzzer**	Move to the next station
	Read instructions
**10 Min → start again**	
**Long Buzzer**	Enter the room, begin the encounter

Comments or new cases

Please contact:

- Dr. Karima Khamisa (Co-Chief Examiner T&T OSCE)
  - kkhamisa@toh.ca
- Dr. Samantha Halman (Co-Chief Examiner Francophone)
  - shalman@toh.ca
- Debra Pugh (Director, Exam Centre)
  - dpugh@toh.ca

Questions?
