# Supplementary material for: The implementation and evaluation of an e-Learning training module for objective structured clinical examination raters in Canada
Source: J Educ Eval Health Prof. 2018 Aug 6;15:18. doi: 10.3352/jeehp.2018.15.18 (PMC6194479; doi:10.3352/jeehp.2018.15.18)
Supplement: Supplementary file 3 — Supplement 3. Participant survey tool. [file jeehp-15-18-suppl3.pdf]

Supplement 3: Survey
1. Which of the following best describes you?
Attending physician (working for 5 years or less)
Attending physician (working for more than 5 years)
PGY-1
PGY-2
PGY-3
PGY-4
PGY-5 or above
2. Number of times you have previously been an OSCE examiner
First time
1-2
3-5
6-10
>10
3. Which format of the examiner orientation do you prefer?
Online orientation
In-person orientation
A combination of both options
No preference
Not applicable (this was my first time as an examiner)
Comments:
4. What was your level of comfort with the scoring task during the OSCE?
Not very confident
Somewhat confident
Confident

Very confident
5. What, if anything, would have helped you be better prepared for the task?
6. Please rate the various aspects of the examiner orientation listed below
Login Process
Instructions on how to navigate the presentation
Length of time required to complete the online training
Clarity of content
Interactivity of the presentation
7. How long did you take to complete this online module?
8. Is there any other feedback you'd like to provide about any elements of the OSCE examiner orientation? Please comment:
